# Enrichment Analysis Identifies Functional MicroRNA-Disease Associations in Humans

**DOI:** 10.1371/journal.pone.0136285

**Published:** 2015-08-21

**Authors:** Dandan Yuan, Xiaomeng Cui, Yang Wang, Yilei Zhao, Huiying Li, Suangjiu Hu, Xiaodan Chu, Yan Li, Qiang Li, Qian Liu, Wenliang Zhu

**Affiliations:** 1 Department of Obstetrics and Gynecology, the Second Affiliated Hospital of Harbin Medical University, Harbin, China; 2 School of Measurement and Control Technology & Communications Engineering, Harbin University of Science and Technology, Harbin, China; 3 Department of Pharmacy, the First Affiliated Hospital of Harbin Medical University, Harbin, China; 4 Department of Obstetrics and Gynecology, Hongqi Hospital of Mudanjiang Medical University, Mudanjiang, China; 5 Department of General Surgery, the Second Affiliated Hospital of Harbin Medical University, Harbin, China; 6 Institute of Clinical Pharmacology, the Second Affiliated Hospital of Harbin Medical University, Harbin, China; Dana-Farber Cancer Institute, UNITED STATES

## Abstract

Substantial evidence has shown that microRNAs (miRNAs) may be causally linked to the occurrence and progression of human diseases. Herein, we conducted an enrichment analysis to identify potential functional miRNA-disease associations (MDAs) in humans by integrating currently known biological data: miRNA-target interactions (MTIs), protein-protein interactions, and gene-disease associations. Two contributing factors to functional miRNA-disease associations were quantitatively considered: the direct effects of miRNA that target disease-related genes, and indirect effects triggered by protein-protein interactions. Ninety-nine miRNAs were scanned for possible functional association with 2223 MeSH-defined human diseases. Each miRNA was experimentally validated to target ≥ 10 mRNA genes. Putative MDAs were identified when at least one MTI was confidently validated for a disease. Overall, 19648 putative MDAs were found, of which 10.0% was experimentally validated. Further results suggest that filtering for miRNAs that target a greater number of disease-related genes (n ≥ 8) can significantly enrich for true MDAs from the set of putative associations (enrichment rate = 60.7%, adjusted hypergeometric *p* = 2.41×10^−91^). Considering the indirect effects of miRNAs further elevated the enrichment rate to 72.6%. By using this method, a novel MDA between miR-24 and ovarian cancer was found. Compared with scramble miRNA overexpression of miR-24 was validated to remarkably induce ovarian cancer cells apoptosis. Our study provides novel insight into factors contributing to functional MDAs by integrating large quantities of previously generated biological data, and establishes a feasible method to identify plausible associations with high confidence.

## Background

MicroRNAs (miRNAs) are a class of small (approximately 22 nt) endogenous non-coding RNA molecules that fine-tune protein abundance via posttranscriptional gene regulation [1,2]. The conserved function of miRNAs, especially the integrative effect of functionally related miRNAs, is an essential strategy that evolved over time to rapidly adjust protein levels in response to biological signals [3]. Over the past decade, the role that miRNAs play in gene regulation has attracted considerable attention in a wide range of fields, including the diagnosis, prevention, and treatment of complex disease [4–6]. Recently, experimental evidence of miRNA-disease associations (MDAs) has been rapidly accumulated; this strongly suggests a wide range of potential clinical applications for miRNAs [7].

Despite rapid progress and constant discovery of new functional MDAs, we are still far away from fully understanding the principles by which miRNAs participate in disease [8]. A systematic analysis performed by Lu et al. established a framework for understanding the association between miRNAs and disease [9]. This study led to the hypothesis that miRNAs with similar functions may tend to influence phenotypically similar diseases. Driven by this hypothesis, many computational prediction methods were developed to identify putative MDAs [10–13]. Meanwhile, such efforts also suggested that more biological data should be taken into account in order to increase prediction accuracy. This strongly implies that other principles underlying functional MDAs remain undiscovered. Inevitably, more in-depth understanding of MDAs will further improve the accuracy of existing methods.

An important question remains unanswered: what is the mechanism by which a miRNA could influence disease? In the present study, we speculated that a true MDA would depend on the contribution of direct and indirect interactions. A miRNA could influence disease progression directly if a miRNA targeted genes related with the disease of interest. Besides direct interaction, a miRNA may also indirectly affect disease phenotypes by regulating the expression of non-disease-related genes (NDGs) that are functionally associated with disease-related genes (DGs). This speculation prompted us to revisit the set of experimentally validated MDAs [7]. In this study, the enrichment of true MDAs was investigated in the reservoir of possible MDAs. Both direct and indirect interactions were considered in conjunction for possible MDAs by counting miRNA-targeted DGs and calculating the protein interaction score (PIS). The PIS was applied to quantitatively assess the extent of association between protein products of miRNA target genes and DGs [14]. Additionally, integration of only experimentally validated data, including MDAs [7], miRNA-target interactions (MTIs) [15], protein-protein interactions (PPIs) [16], and gene-disease associations (GDAs) [17] helped to control the false positive rate in this analysis [11]. By applying this approach, miR-24 was identified for the first time to be functionally associated with ovarian cancer. In vitro experiments validated that transfection of miR-24 indeed resulted in significant apoptosis of ovarian cancer cells, compared to the effect of scramble miRNA transfection as negative control. In summary, the above efforts aim to reveal the composition of factors responsible for MDAs, and to identify putative novel MDAs.

## Materials and Methods

### Data acquisition and processing

To characterize functional MDAs, biological information from multiple sources was collected in this present study. The entire dataset of experimentally validated MDAs were retrieved from the Human microRNA Disease Database (HMDD) database version 2.0 [7]. Next, each disease name was confirmed on the Medical Subject Headings (MeSH) website (http://www.ncbi.nlm.nih.gov/mesh/); only MDAs with MeSH-defined disease names were retained, and annotated with their MeSH tree numbers (S1 Table). In addition, for a portion of the MDAs, it was necessary to combine the annotations according to the mature miRNA sequence ID in miRBase [18]. For example, the MDAs caused by hsa-let-7a-1, hsa-let-7a-2 or hsa-let-7a-3 were all attributed to the parent miRNA, hsa-let-7a. The miRTarBase database was used to obtain the set of experimentally supported MTIs in humans [15]. Notably, only the MTIs supported by Strong Evidence or ≥ 2 pieces of Less Strong Evidence (as defined by miRTarBase) were trusted and retained (S2 Table). BisoGenet, a Cytoscape [19] plug-in, was used to retrieve human PPIs from multiple public datasets, including the Biomolecular Interaction Network Database (BIND, http://bind.ca), the Biological General Repository for Interaction Datasets (BioGRID, http://thebiogrid.org/), the Database of Interacting Proteins (DIP, http://dip.doe-mbi.ucla.edu/dip/Main.cgi), the IntAct Protein Interaction Database (http://www.ebi.ac.uk/intact/), the Molecular INTeraction database (MINT, http://mint.bio.uniroma2.it/mint/), and the Human Protein Reference Database (HPRD, http://www.hprd.org). Only the PPIs that were experimentally validated by yeast two-hybrid assays were included in this study. The database of gene-disease associations (DisGeNET) was queried to obtain high confidence GDAs, identified as those GDAs that pass the DisGeNET score threshold of 0.2 [17]. As described for the MDAs, only the GDAs with MeSH-defined disease names were retained (S3 Table). Both gene symbols and Entrez gene IDs were recorded to facilitate further analysis. All genes, including miRNA target genes and DGs studied here, were protein-coding genes [20].

### MTI counting and PIS calculation

In this study, we considered the number of MTIs as a measure of direct effects that may result in a putative MDA. For this purpose, the lists of MTIs and GDAs were imported into the bioinformatics software Cytoscape and integrated to build a miRNA-gene-disease network (MGDN). Genes represented as nodes were deleted from the network if they were not simultaneously connected to a miRNA and a disease. For each miRNA in the MGDN, the topological parameter degree was calculated to count the number of MTIs for each disease. Putative functional MDAs were identified where at least one MTI existed between a miRNA and a disease. Only the miRNAs targeting at least 10 mRNA genes were included in the MGDN.

In our previous work, we established a PIS parameter to quantitatively assess the extent of association between protein products of genes targeted by different miRNAs [14]. High PIS implies dense interactions between proteins regulated by different miRNAs. We applied the PIS parameter in the present study as a quantitative measure of indirect effects that may result in a putative MDA. Suppose a MDA that is composed of miRNA *i* and disease *j*. To calculate PIS, the proteins encoded by the target genes of miRNA *i* and the DGs of disease *j* should be divided into three clusters in advance. Cluster 1 contains the proteins that are only regulated by miRNA *i*, cluster 2 accommodates those that are only encoded by the DGs of disease *j*, and cluster 3 is composed of the proteins that are both regulated by miRNA *i* and encoded by the DGs of disease *j*. After retrieving experimentally-validated human PPIs from multiple public datasets by using BisoGenet [16], PIS was calculated as the sum of proteins divided by the sum of PPIs between clusters, as defined before [14]. Due to large number of MDAs analyzed here, a custom computer program written in Java was designed for batch calculation of PIS (S1 File). Before PIS calculation, four data files should be prepared, which contain the sets of experimentally-validated MTIs, confidently-trusted GDAs, experimentally validated PPIs between proteins encoded by miRNA target genes and DGs, and true MDAs recorded in the HMDD database, respectively.

### Hypergeometric enrichment analysis

The authenticity of all MDAs in the MGDN was verified according to the experimental evidence extracted from the HMDD database. Briefly, MDAs were marked as ‘true’ if evidence was found to confirm the miRNA-disease association. Enrichment rate was defined as the percentage of true MDAs among a given set of putative functional MDAs. High enrichment rate of true MDAs implies that there be a greater chance to identify putative novel MDAs among the given set of MDAs. The GeneProf hypergeometric probability calculator (http://www.geneprof.org/GeneProf/tools/hypergeometric.jsp) was then applied to evaluate whether true MDAs were over-represented in the total number of possible MDAs at different MTI threshold counts, with or without PIS restriction. The significance of true MDA enrichment was determined, and the hypergeometric *p*-values were adjusted for multiple comparisons by the Benjamini and Hochberg method of multiple testing correction [21]. Adjusted *p* values of *p* < 0.05 were considered significant.

### Cell culture and miRNA transfection

The human ovarian cancer cell lines SKOV3 (p53 mutant-type) and A2780 (p53 wild-type) were purchased from Tianjin Life Science Research Center and Shanghai BoQuaner biological science and technology company, respectively. Briefly, cells were cultured in 10% fetal bovine serum (FBS)-RPMI 1640 in a 37°C, 5% CO_2_ humidified incubator as described before [22]. Transfection of miR-24 was performed by using X-treme GENE (Roche, Swiss), according to the procedure specification. Three transfection concentrations (25, 50 and 100 nM) of miR-24 were investigated of potential effect on cell injury in SKOV3 cells with scramble miRNA as negative control at different time points, respectively. The concentration of 50 or 100 nM was applied for miR-24 transfection in the A2780 cells. Furthermore, the miR-24 inhibitor (anti-miRNA oligonucleotide of miR-24, AMO-24) was used to be co-transfected with miR-24 to validate the specificity of action of the latter.

### Cell viability assay

Cell viability was assessed by measuring mitochondrial dehydrogenase activity, using the colorimetric MTT assay, based on the fact that viable cells (but not dead cells) can reduce 3-(4,5-dimethylthiazol-2-yl)-2,5-diphenyl tetrazolium bromide (MTT, Amrsco, USA). After miRNA transfection, cells were incubated with 10 mL MTT of 0.5 mg/ml at 37°C for 48 h. The purple formazan crystal was dissolved with 150 μL of dimethyl sulfoxide (DMSO, Amrsco, USA) and added to the cells. The absorbance was measured at 490 nm.

### TUNEL-staining assay

After transfection of 100 nM miR-24 or scramble miRNA, cells were cultured for 48 h. Briefly, cells were fixed with 4% paraformaldehyde and permeabilized with 0.1% Triton X-100 sodium citrate buffer after washing 3 times with phosphate buffered saline (PBS). An *in situ* cell death detection kit (Roche, San Francisco, CA, USA) was then applied to label apoptotic cells, and the nuclei were stained with 4',6-diamidino-2-phenylindole (DAPI). The Image-Pro Plus software (Media Cybernetics Inc., Rockville, MD, USA) was used to count the total number of cells and the TUNEL-positive cells for calculating the apoptosis rate that was defined as the ratio of apoptotic cells to total cells.

### Flow cytometry assay

Cells were assessed by the Annexin V-FITC apoptosis kit (Beyotime institute of Biotechnology, China) according to the manufacturer’s instructions. After treatment, cells were digested, washed, and resuspended in 195 μL binding buffer. The samples were subsequently incubated with 5 μL of Annexin V-FITC and 10 μL of propidium iodide for 12 min at room temperature in the dark and then analyzed by flow cytometry (BD Facs Canto II, USA).

### Western blotting assay

Protein samples were isolated from A2780 cells. Briefly, the cells were seeded in a 6-well plate at 37°C in 5% CO_2_. After miRNA transfection and cell incubation for 48 h, the cells were collected from the plate, and the resulting cell suspension was centrifuged (800×g, 10 min, 4°C). The cell pellets were ultrasonicated for 15 min (every 15 s with 5 min intervals) at 4°C in cell lysate buffer (RIPA buffer: 50 mM Tris pH 7.4, 150 mM NaCl, 1% Triton X-100, 1% sodium deoxycholate, 0.1% SDS, sodium orthovanadate, sodium fluoride, EDTA and leupeptin). Finally, the lysed cells in buffer were centrifuged at 1000×g for 15 min, and the supernatant (protein) samples were kept for Western blotting. The isolated protein samples were subjected to 15% SDS-polyacrylamide gel electrophoresis, blotted onto a nitrocellulose membrane, and then blocked with 5% non-fat milk for 120 min. Next, the membranes were probed with anti-*p*-MDM2 ((phospho-mouse double minute 2, Ser 166) (1:1000 dilution ratio, Abcam Inc.), anti-CDK4 (cyclin-dependent kinase 4) (1:1000 dilution ratio, Abcam Inc.) and anti-p53 (1:1000 dilution ratio, Abcam Inc.) in phosphate-buffered saline (PBS) containing 1% BSA and incubated overnight at 4°C. Thereafter, membranes were washed three times with PBS for 30 min and incubated with horseradish peroxidase-labelled secondary antibody for 1 h. GAPDH (1:1000 dilution ratio, Kangcheng Inc, China) was used as an internal control. The bands were acquired using an imaging system (LI-COR Biosciences; Lincoln, NE, USA). Odyssey v3.0 software was used to measure the band intensity [area×optical density (OD)] in each group.

### Patient recruitment and tissue sample collection

This study was approved by the Ethics Committee of Harbin Medical University. Each patient enrolled in this study was informed of the project and had signed a written consent. Twelve patients with ovarian cancer were recruited in this study, which were in an age range from 43 to 68. Among them, one patient was in the I stage, two in the II stage, and nine in the III stage according to the International Federation of Gynecology and Obstetrics (FIGO) staging system [23]. Pathological test after surgery confirmed that all the cancers were derived from serous or mucinous epithelial tumors. Cancerous ovarian tissue of about 1.0 cm in diameter was obtained from each patient with ovarian cancer. Additionally, this study also included ten patients with normal ovary that was pathologically confirmed of non-cancerous lesion. All of the ten patients were in an age range from 42 to 62 and subjected to ovariectomy due to uterine disease, from each of which normal ovarian tissue of about 0.5 cm was obtained during surgery. All the tissues were stored in liquid nitrogen before quantitative reverse transcription–polymerase chain reaction (qRT-PCR) assay. No significant difference in age was found between the patients with ovarian cancer and the participants without cancerous lesion in ovary.

### qRT-PCR assay

Briefly, total RNA was extracted using Trizol reagent, RNA extracts of 0.5 μg was reverse transcribed into cDNA in 10 μL reactions using a high-capacity cDNA reverse transcription kit (Applied Biosystems, USA). For real-time PCR, 2×SYBR Green PCR Master Mix was used according to the manufacturer’s instructions. qRT-PCR assay was performed on ABI 7500 fast Real Time PCR system (Applied Biosystems, USA). After a brief heating (10 min, 95°C), amplification parameters were as followed: 95°C for 15 s, 60°C for 1 min, 40 cycles. Cycle threshold (CT) values of miR-24 were normalized to U6 and calculated by using the following the equation of 2^−ΔΔCT^ as described before [24].

### Statistical analysis

All data are expressed as mean ± SEM. Statistical analysis was performed using student's *t* test or one-way ANOVA followed by Tukey's test, where appropriate. Differences were considered statistically significant when *p* < 0.05.

## Results

**Fig 1:**
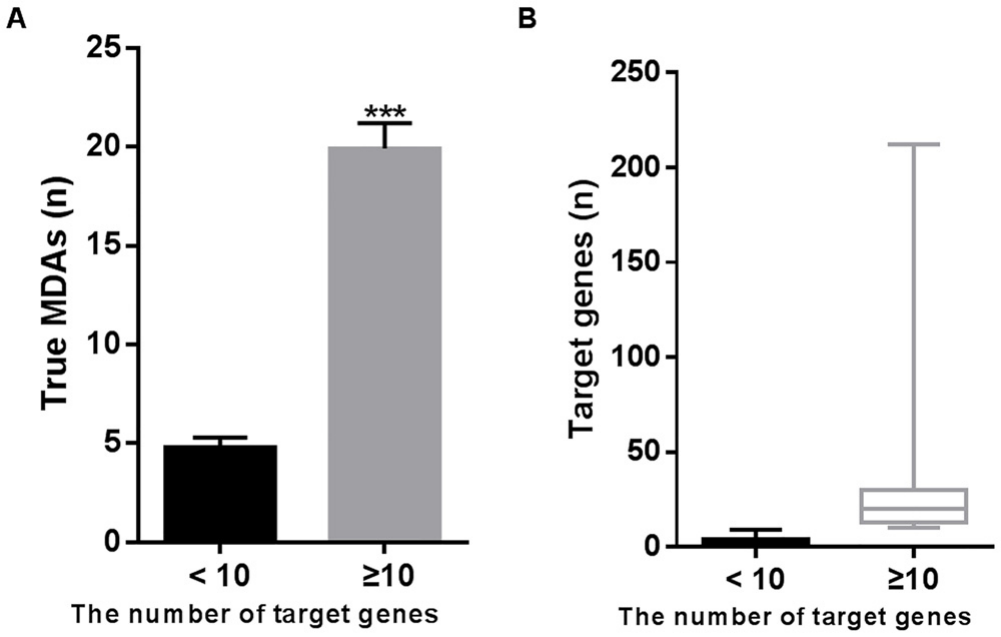
Comparison of the number of true MDAs between miRNAs with < 10 target genes and those with ≥ 10 target genes. (**A**) MiRNAs with ≥ 10 target genes are involved with more true MDAs than those targeting less target genes. *** *p* < 0.001 versus < 10; (**B**) Box-and-whisker plots of the number of target genes for the two groups.

In total, 11844 protein-coding genes were found to be related with at least one of the 2223 MeSH-defined human diseases, and constituted 51007 GDAs that were experimentally validated (S3 Table). A high-confidence set of 3536 MTIs was established between 347 miRNAs and 1877 protein-coding genes in humans (S2 Table). Among the 347 miRNAs, 99 were experimentally confirmed to target at least 10 mRNA genes. Although they account for only 28.5% of the total miRNAs, 2862 MTIs was assigned to them (approximately 81%). Due to targeting more mRNA genes, they were found to be implicated in more true MDAs than those targeting less target genes (Fig 1). By integrating the above data into a MGDN, 19648 putative MDAs were identified for them (see Materials and Methods, S1 Fig). The putative MDAs were then scanned for supporting experimental evidence in the HMDD (S1 Table). Approximately 10% of the MDAs (n = 1971) were verified as true occurrences in humans. Finally, the number of MTIs and the PIS were counted and calculated for each putative MDA, respectively.

### True MDAs tend to be caused by miRNAs that directly target more DGs

**Fig 2:**
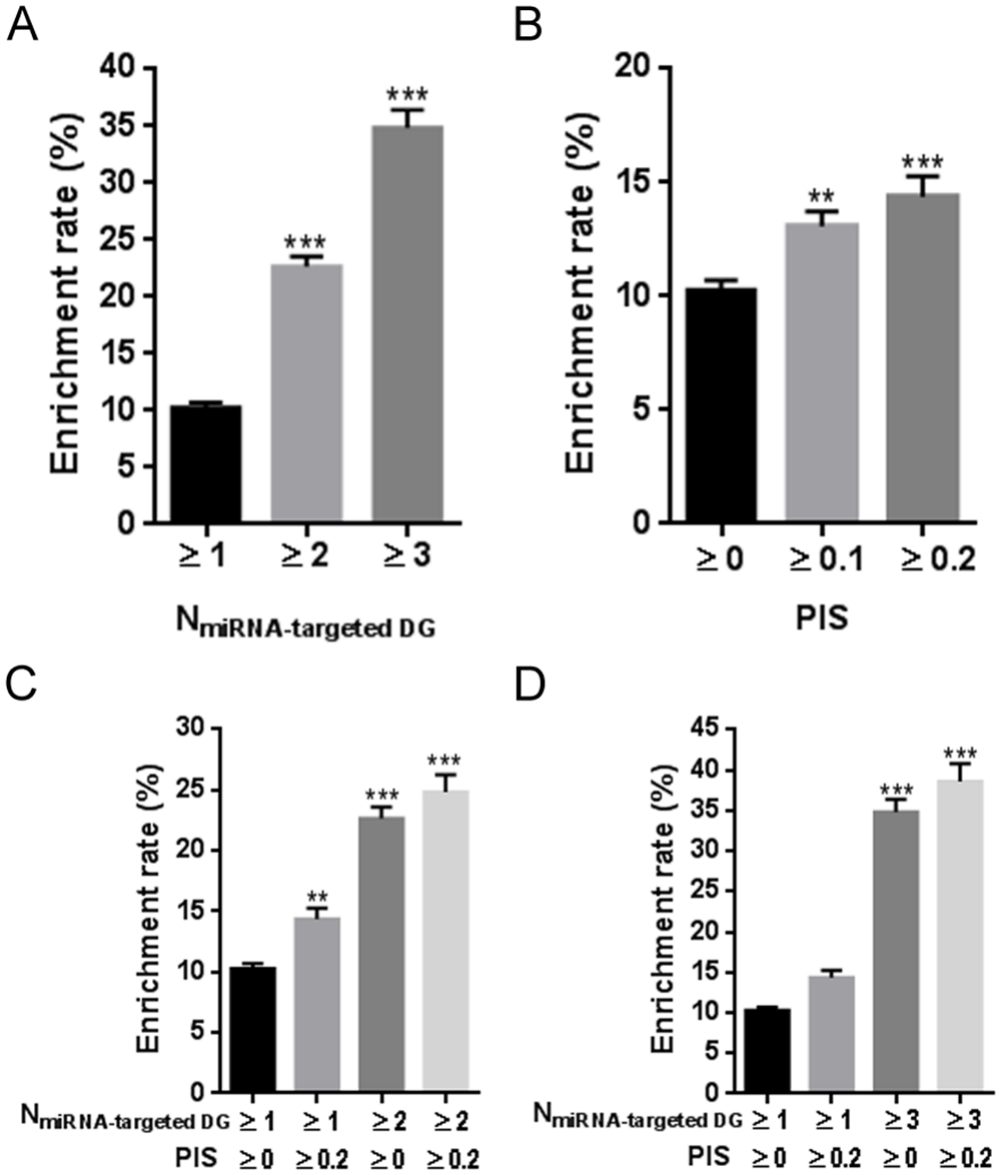
Average effect of direct and indirect interactions on enrichment of experimentally validated MDAs. (**A)** Increasing the number of miRNA-targeted DGs enriches for true MDAs. *** *p* < 0.001 versus ≥ 1; (**B)** Denser protein-protein association between miRNA-targeted NDGs and DGs tends to facilitate true MDAs. ** *p* < 0.01, *** *p* < 0.001 versus ≥ 0; (**C** and **D**) Effect on true MDA enrichment by co-consideration of both factors. ** *p* < 0.01, *** *p* < 0.001 versus the N_miRNA-targeted DGs_ ≥ 1 and PIS ≥ 0 group; N_miRNA-targeted DGs_: the sum of DGs that are targeted by a given miRNA. MDA: miRNA-disease association; NDG: non-disease-related gene; DG: disease-related gene.

**Fig 3:**
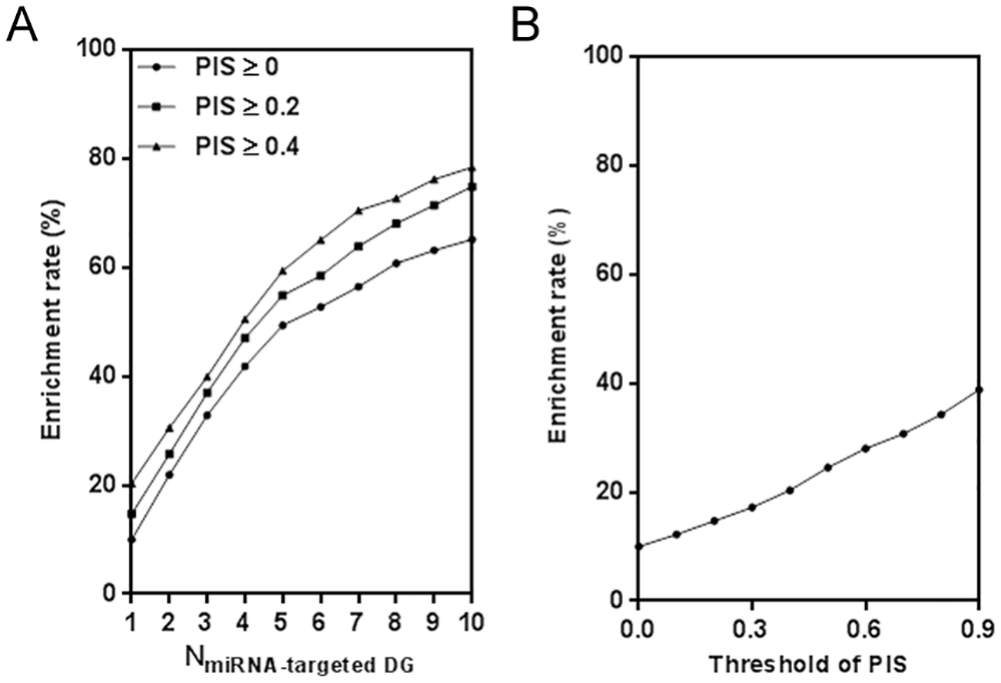
Overall effect of direct and indirect interactions on enrichment of experimentally validated MDAs. (**A**) Increasing the number miRNA-targeted DG enrichments for true MDAs in the whole set of putative MDAs. (**B**) Comparably, higher PIS value only slightly enriches for true MDAs. MDA: miRNA-disease association; DG: disease-related gene; PIS: protein interaction score.

In this study, the direct effects factor assumed that each of the 19648 possible MDAs possessed at least one MTI. It was then investigated whether the number of MTIs could be used to identify true MDAs. This analysis revealed that miRNAs that target a greater number of DGs significantly increased the possibility that a given MDA may be real (Fig 2A, *p* < 0.001). This finding was confirmed after the entire set of 99 miRNAs was analyzed together (Fig 3A). Approximately two-thirds of the possible MDAs were assigned using only one MTI. Among them, only 616 MDAs were verified by experimental evidence in HMDD. In comparison, limiting the selection of putative MDAs to those that possessed a greater number of MTIs was effective in enriching for true MDAs. More than two-thirds of the real MDAs were assigned to those with ≥ 2 MTIs (n = 6185). Furthermore, if a strict threshold of ≥ 6 MTIs was used, experimental evidence in the HMDD database could be found for more than half of the MDAs (enrichment rate = 52.7%, hypergeometric adjusted *p* = 3.96×10^−163^). The MDAs with ≥ 10 MTIs are listed in S4 Table, of which 65.1% were validated by HMDD. Our findings suggest that the sum of diseased-related target genes may be an important contributing factor to functional MDAs.

### Indirect gene regulation further highlights the effect of miRNAs on human disease

Considering the complexity of human signaling networks, we also investigated how allowing indirect interactions might contribute to identifying functional MDAs. We scored the number of indirect interactions by calculating the PIS. Our findings indicate that indirect gene regulation should not be ignored, despite its smaller contribution to identifying true MDAs than direct interactions between miRNAs and their target DGs (Figs 2B and 3B). Comprehensive consideration of both direct and indirect interactions increased the enrichment rate of true MDAs (Fig 2C and 2D). Overall, the success of indirect interactions to predict true MDAs further highlights the effect of miRNAs on disease (Fig 3A). For instance, the initial enrichment rate of true MDAs was 60.7% from the set of putative MDAs with ≥ 8 MTIs. However, an ultimate enrichment rate of nearly 73% could be achieved if indirect interactions were also considered. In particular, the MDAs with ≥ 6 MTIs and PIS of ≥ 0.4 are highlighted in S4 Table, of which 65.0% were validated by HMDD.

### Literature search validates functional MDAs with ≥ 10 MTIs and PIS of ≥ 0.4

As consideration of both direct and indirect interactions enriched for true MDAs, we speculated that MDAs with greater numbers of MTIs and higher PIS values might be more functionally associated than those with fewer MTIs and lower PIS values. To test this hypothesis, a literature search was performed for the MDAs with the highest degree of association, as measured by the number of MTI and the PIS value. By using the names of the miRNA and disease as joint search terms, a PubMed literature search was performed for the 18 MDAs that we identified with ≥ 10 MTIs and with PIS values ≥ 0.4. Unlike the other 65 MDAs with ≥ 10 MTIs and PIS of ≥ 0.4, these 18 MDAs were not found in the HMDD database (S1 and S4 Tables). Table 1 lists the search results that show experimental evidence for 11 of the 18 total MDAs [25–36]. This finding supports the effectiveness of our enrichment analysis in identifying putative MDAs.

**Table 1:** Results of literature search for 18 highly plausible MDAs.

MDA	N_miRNA-targeted DG_	PIS	experimental evidence	Reference
miR-16:Alzheimer Disease	16	0.426	Overexpression of miR-16 reduces amyloid protein precursor expression	[25]
miR-16:Diabetes Mellitus, Type 2	11	0.413	Overexpression of miR-16 impairs circulating proangiogenic cell functions	[26]
miR-16:Lung Neoplasms	17	0.732	cell cycle regulation	[27]
miR-16:Pulmonary Disease, Chronic Obstructive	12	0.771	N/A	-
miR-16:Urinary Bladder Neoplasms	16	0.692	cell proliferation	[28]
miR-21:Asthma	13	0.428	overexpressed in patients with asthma	[29]
miR-21:Neoplasm Metastasis	12	0.990	promoting cancer metastasis	[30,31]
miR-21:Pulmonary Disease, Chronic Obstructive	17	0.890	prognostic biomarker	[32]
miR-24:Colorectal Neoplasms	12	0.810	cell proliferation	[33]
miR-24:Ovarian Neoplasms	11	1.110	N/A	-
miR-24:Pulmonary Disease, Chronic Obstructive	11	0.850	N/A	-
miR-145:Pulmonary Disease, Chronic Obstructive	13	0.538	N/A	-
miR-146a:Pulmonary Disease, Chronic Obstructive	10	0.484	abnormal inflammatory response	[34]
miR-155:Alzheimer Disease	16	0.433	N/A	-
miR-155:Diabetes Mellitus, Type 2	14	0.461	dysregulated expression in patients with type 2 diabetes	[35]
miR-155:Myocardial Ischemia	12	0.402	atherosclerotic inflammatory responses	[36]
miR-155:Prostatic Neoplasms	18	0.457	N/A	-
miR-155:Pulmonary Disease, Chronic Obstructive	17	0.643	N/A	-

Result of literature search for 18 highly plausible MDAs. MDA: miRNA-disease association; DG: disease-related gene; PIS: protein interaction score; N/A: not available.

### Co-consideration of MTI and PIS identifies functional MDAs in complex human diseases

**Fig 4:**
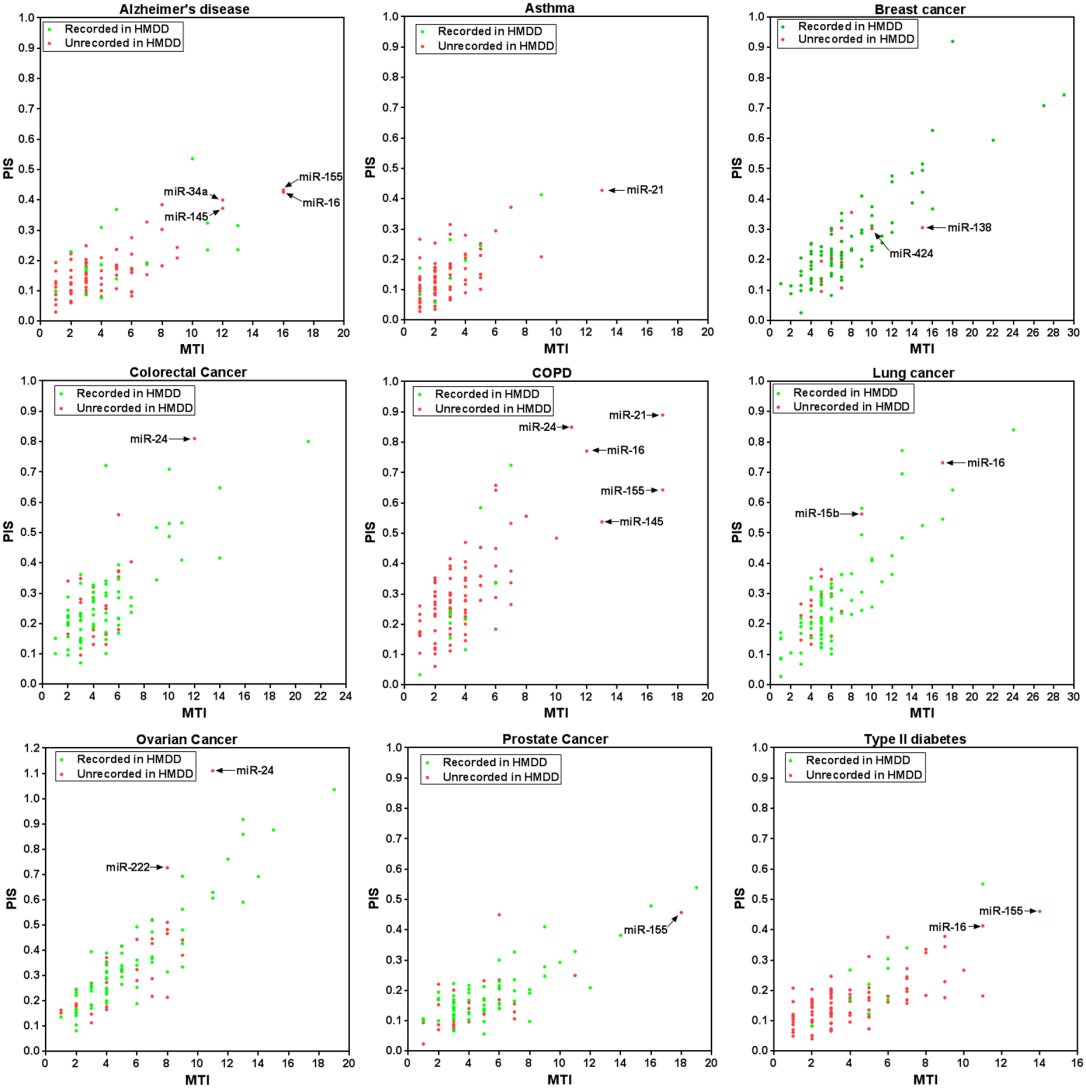
Identification of functional MDAs for nine complex human diseases by co-considering direct and indirect interactions. Highly plausible MDAs were highlighted. COPD: chronic obstructive pulmonary disease.

In this study, nine complex human diseases were exampled for illustrating the possible application of our method to identify functional MDAs in humans. Our result indicates that four diseases [Alzheimer's disease (AD), asthma, chronic obstructive pulmonary disease (COPD), and type II diabetes (T2D)] relatively lack of experimental evidence of functional MDAs. Fig 4 intuitively indicated miRNAs that be most probably associated with them. For instance, despite unrecorded in HMDD, high MTI and PIS strongly suggested functional involvement of miR-16 in AD, COPD, lung cancer, and T2D. This is consistent with previous functional studies [25–27]. There is no study reporting any functional role of miR-16 in COPD. Evidence has shown that miR-21 be overexpressed with patients with asthma [29]. Our study suggests that miR-21 may be functionally implicated in asthma.

**Fig 5:**
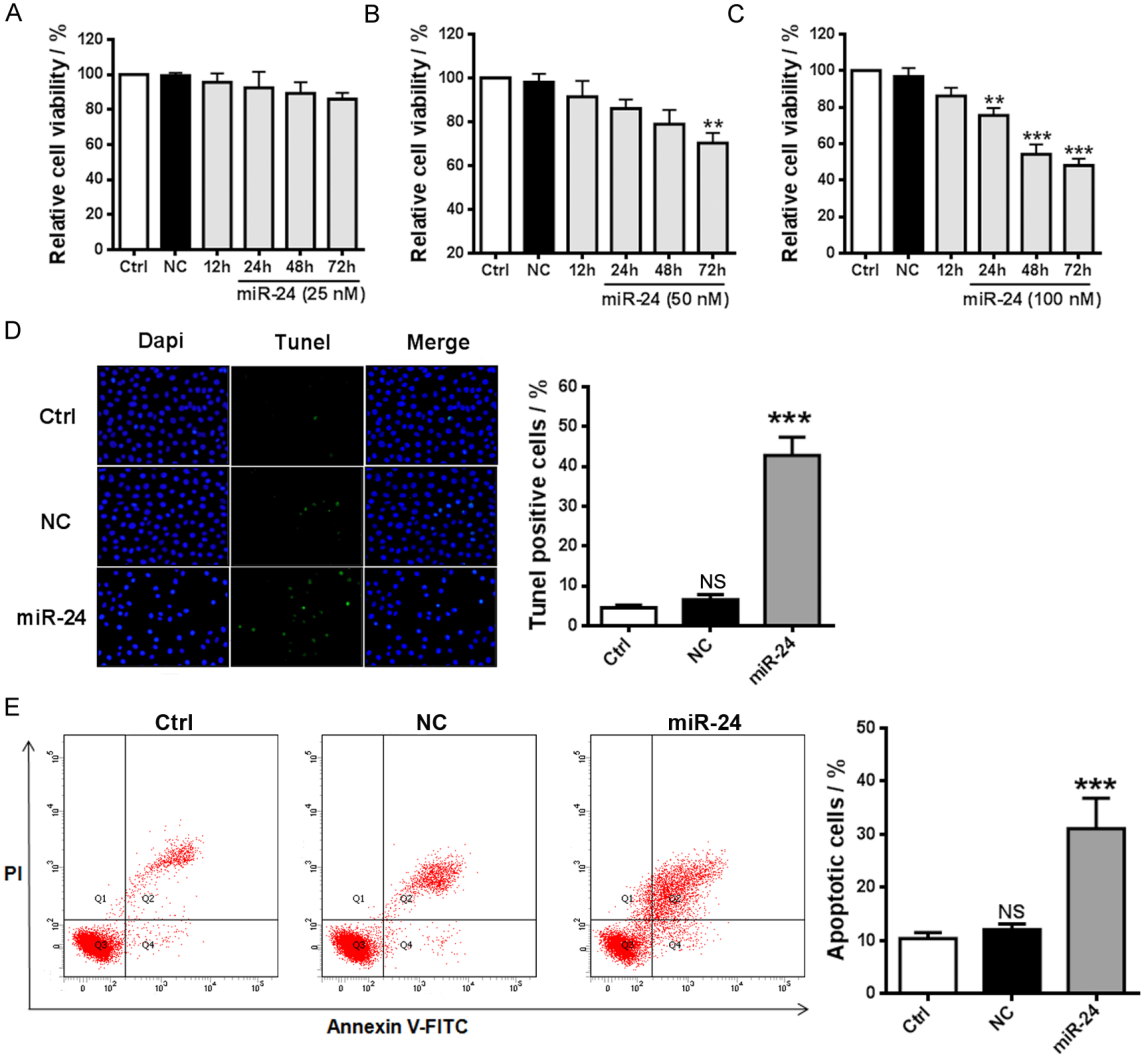
Pro-apoptotic effect of miR-24 in SKOV3 cells. (**A**, **B** and **C**) Effect of transfection of different concentrations of miR-24 on cell viability of SKOV3 cells (n = 7). ** *p* < 0.01, *** *p* < 0.001 versus NC; (**D**) Transfection of 100 nM miR-24 induced cell apoptosis of SKOV3 cells (TUNEL assay, n = 5). *** *p* < 0.001 versus NC. (**E**) Transfection of 100 nM miR-24 resulted in cell apoptosis of SKOV3 cells (flow cytometry assay, n = 3). *** *p* < 0.001 versus NC. NC: negative control cells that were transfected with scramble miRNA.

**Fig 6:**
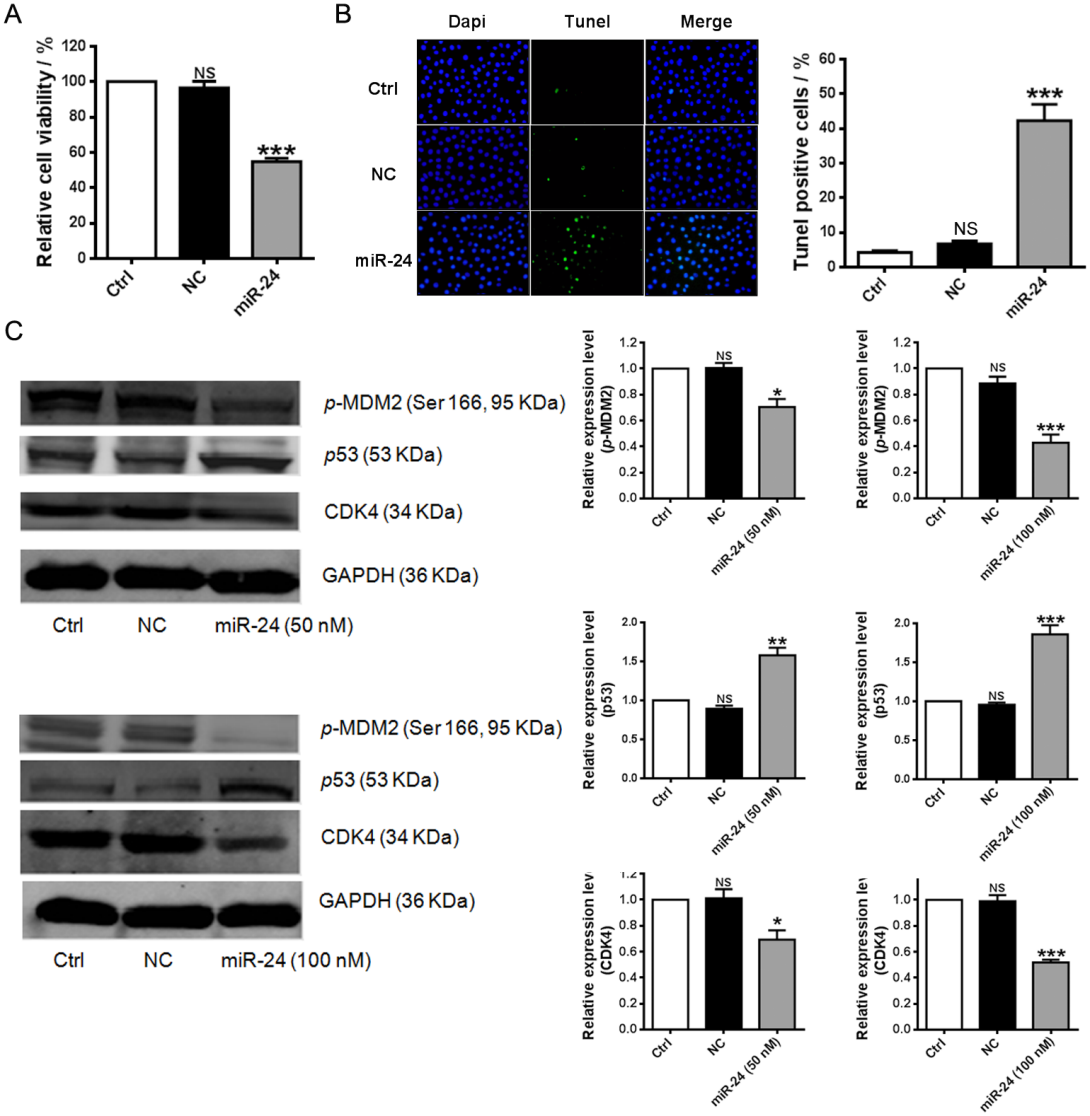
Pro-apoptotic effect of miR-24 in A2780 cells. (**A)** Effect of transfection of 100 nM miR-24 on cell viability of A2780 cells (n = 7). *** *p* < 0.001 versus NC; (**B**) Transfection of 100 nM miR-24 induced cell apoptosis of A2780 cells (TUNEL assay, n = 5). *** *p* < 0.001 versus NC. (**C**) Transfection of miR-24 reduced the CDK4 and *p*-MDM2 protein level but enhanced the expression of p53 (Western blot assay, n = 3). ** *p* < 0.05, ** *p* < 0.01, *** *p* < 0.001 versus NC. NC: negative control cells that were transfected with scramble miRNA; NS: No significant difference was found between Ctrl group and NC group.

Additionally, in vitro experiment was performed here to investigate potential effect of miR-24 in ovarian cancer. Cell viability assay indicates that compared with low transfection concentrations 100 nM of miR-24 significantly induce cell apoptosis of the p53 mutant-type ovarian cancer cells (Fig 5A–5C, *p* < 0.001; S2A Fig). This finding was further confirmed by the measurement of cell apoptosis with TUNEL and flow cytometry assays (Fig 5D and 5E), suggesting obvious pro-apoptotic role of miR-24 in the ovarian cancer cells. It was also found that overexpression of miR-24 remarkably led to cell apoptosis of the p53 wild-type ovarian cancer cells (Fig 6A and 6B; S2B Fig). Cotransfection with AMO-24 was found to obviously reverse the pro-apoptotic role of miR-24 (S3 Fig). It was further validated that reduced CDK4 and *p*-MDM2 protein level and increased p53 expression in a dose-dependent manner might be involved in the mechanism by which miR-24 promoted cell apoptosis of the ovarian cancer cells (Fig 6C). Obvious downregulation of miR-24 in cancerous ovarian tissues was observed, implying pathological implication in ovarian cancer (S4 Fig).

## Discussion

A typical MDA study can be summarized as a generic workflow, as discussed by De and his colleagues [37]. First, high-throughput techniques are used to identify dysregulated miRNAs in the disease of interest. Next, *in vitro* or *in vivo* experiments are performed to investigate the functional roles of the miRNAs and their target proteins. Eventually, functionally associated MTIs will be validated to illustrate the potential molecular mechanism by which a miRNA may affect disease phenotypes. After a decade of rapid MDA investigation by this technique worldwide, more than 5000 true miRNA-disease associations can be retrieved online today [7]. Promising therapeutic targets and clinical markers are hidden in this MDA reservoir [3–6]. The fact that evidence can be accumulated so rapidly also raises a fundamental issue: what inherently leads to the true occurrence of functional MDAs on such a broad scale [7]. In this study, we proposed that direct interactions between miRNAs and disease-related genes, as well as indirect effects between miRNA targets and disease-related genes could be two sources of functional miRNA-disease associations. We hypothesized that by targeting more DGs, and by having a more widespread impact on DGs through secondary interactions, a miRNA may act as a vital effector in human disease. To test this hypothesis, a MDA enrichment analysis was performed on the basis of biological evidence integration [7,15–17].

As assumed before, the majority of the true MDAs were found to be accompanied by multiple disease-related MTIs. Although one miRNA may potentially regulate hundreds of target genes [15], and many genes may be simultaneously associated with a disease [17], the hypergeometric probability calculation indicated a significant association between true MDAs and the number of MTIs, rather than random occurrence. For instance, 65.1% of the MDAs that possess greater than 10 MTIs were verified as true associations (adjusted hypergeometric *p* = 4.48×10^−60^). In comparison, only 4.6% of MDAs that were supported by just one MTI were true. This finding implies potential authenticity of a MDA if multiple miRNA interactions with disease-related genes could be validated for the involved miRNA and the disease of interest. Our enrichment analysis reveals that the number of miRNA-targeted disease related genes should be seriously considered as an important contributing factor when predicting plausible MDAs.

Comparatively, a miRNA's indirect effect on disease should be assigned with a smaller weight for identification of plausible MDA. Our results reveal that a miRNA may also affect disease phenotypes by regulating the expression of non-disease-related genes that are functionally associated with disease-related genes. However, additional enrichment of true MDAs should not be expected with an increase in PIS score. This finding implies that miRNAs that act primarily through indirect gene regulation and have a small number of validated disease-related target genes have a limited capacity to alter disease phenotypes. Our results agree with this implication. Only 38.8% of MDAs with one MTI and a PIS value ≥ 0.9 have been experimentally validated. The threshold of 0.9 was high for PIS as there were merely 85 such MDAs among all of the putative ones. Despite this limited effect, we found that consideration of direct and indirect factors together gave the best enrichment of true MDAs. Of the 83 putative MDAs with ≥ 10 MTIs and PIS of ≥ 0.4, 78.3% of them had experimental evidence recorded in the HMDD database. However, if the direct effect was considered alone, only 65.1% of MDAs were true. The HMDD-unrecorded MDA miR-24:ovarian cancer was in vitro validated that overexpression of miR-24 shown remarkable pro-apoptotic effect in the human ovarian cancer cells. Consistent with the previous study [33], this finding suggests that miR-24 may play important role as a tumor suppressor in the ovarian cancer cells. It was previously validated that CDK4 was a key regulator in promoting ovarian cancer cells proliferation [38], and specific inhibition of MDM2, a crucial negative regulator of p53, resulted in ovarian cancer cells apoptosis [39]. Our result implies that lowering the expression level of CDK4 and *p*-MDM2 may partly explain for the remarkable pro-apoptotic role of miR-24 and the miR-24-increased p53 expression in the ovarian cancer cells observed here. Furthermore, downregulated expression of miR-24 was validated in human ovarian cancer in the present study. This result should be cautiously considered as the small sample size of participants recruited in this study. Taken together, our results suggest putative MDA between miR-24 and ovarian cancer. Nevertheless, in vivo experimental study is definitely needed for further confirming our results.

In conclusion, our study provides a comprehensive scan of putative MDAs in humans for the first time. By only including experimental data, we believe that our findings are of high confidence. Novel insights were provided for intrinsic factors that contribute to true association between miRNAs and disease. In general, confidence in true MDAs increases with the number of MTIs and the PIS value. Our results strongly suggest that these two quantitative factors should be considered in future studies that aim to predict putative MDAs, or studies that aim to functionally validate miRNA-disease interactions.

## Supporting Information

S1 Fig MiRNA-gene-disease network established in this study. In the network, miRNAs, genes, and diseases represent as blue, green, and red nodes, respectively. Edge indicates experimentally-validated functional association between gene and miRNA/disease.
(TIF)

S2 Fig Validation of miR-24 transfection in SKOV3 cells (A) and A2780 cells (B). NC: negative control cells that were transfected with scramble miRNA; ns: not significant; *** *p* < 0.001; n = 5.
(TIF)

S3 Fig Cotransfection of AMO-24 (50 nM) reversed the pro-apoptotic effect of miR-24 (50 nM) in SKOV3 cells (A) and A2780 cells (B). NC: negative control cells that were transfected with scramble miRNA; ns: not significant; *** *p* < 0.001 versus NC; ^#^ *p* < 0.05, ^###^ *p* < 0.001 versus miR-24, n = 5.
(TIF)

S4 Fig Detection of miR-24 expression in human cancerous (n = 12) and normal (n = 10) ovarian tissues. *** *p* < 0.001 versus normal.
(TIF)

S1 File A custom program designed for PIS calculation. (RAR)

S1 Table Experimentally validated MDAs used in this study. (XLSX)

S2 Table Experimentally validated MTIs used in this study. (XLSX)

S3 Table Experimentally validated GDAs used in this study. (XLSX)

S4 Table MDAs with highest degree of significance. (XLSX)
