# Supplementary figures and images for: Enrichment Analysis Identifies Functional MicroRNA-Disease Associations in Humans
Source: PLoS One. 2015 Aug 21;10(8):e0136285. doi: 10.1371/journal.pone.0136285 (PMC4546424; doi:10.1371/journal.pone.0136285)

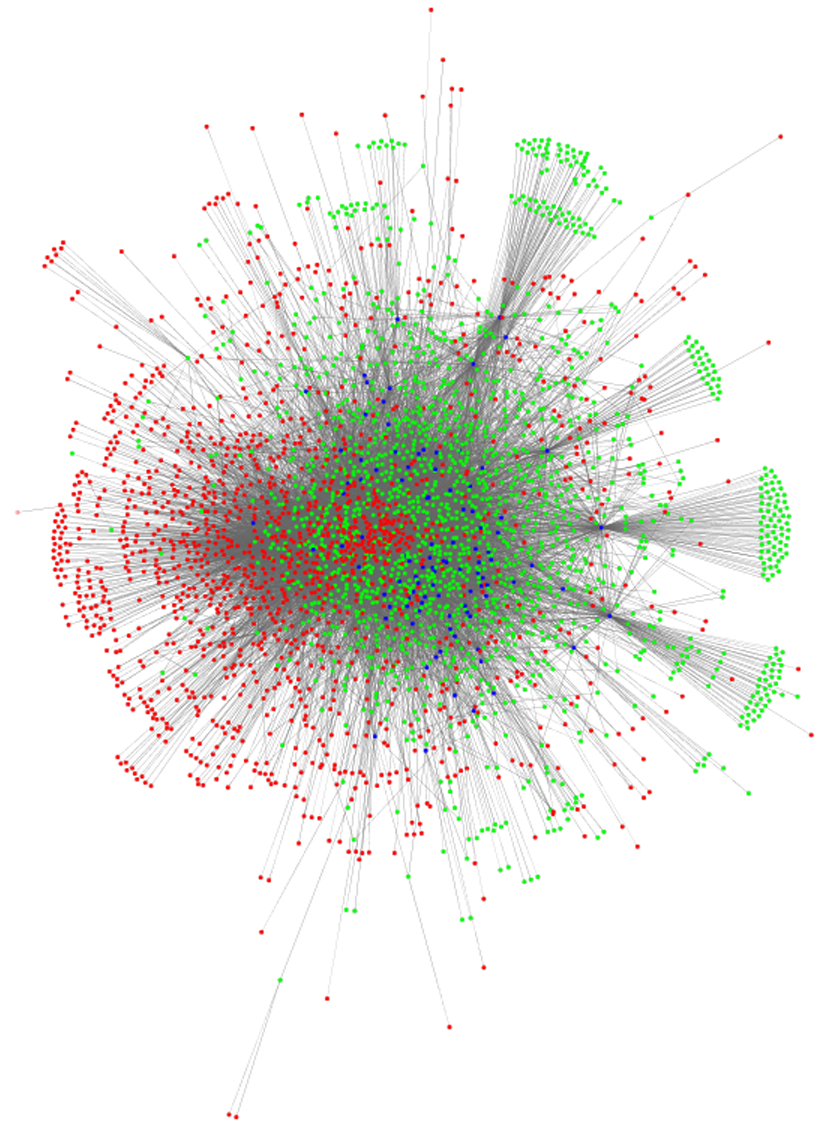

Supplement: S1 Fig — In the network, miRNAs, genes, and diseases represent as blue, green, and red nodes, respectively. Edge indicates experimentally-validated functional association between gene and miRNA/disease. (TIF) [file pone.0136285.s001.tif]

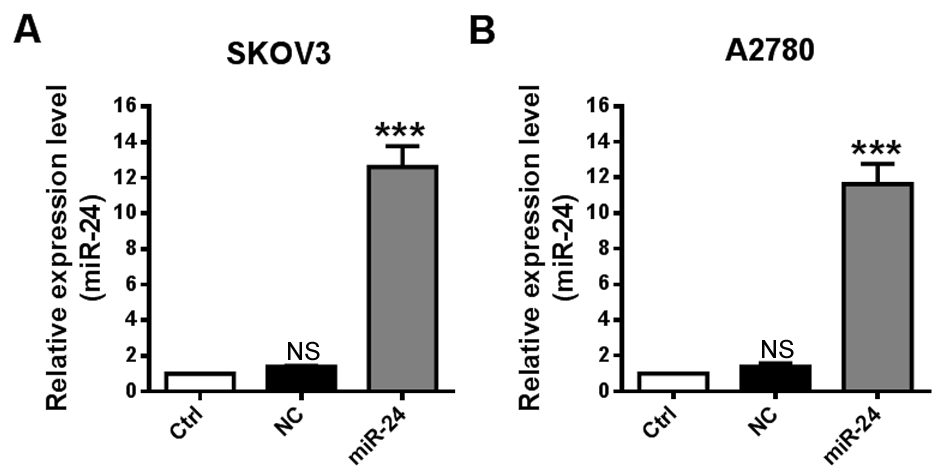

Supplement: S2 Fig — NC: negative control cells that were transfected with scramble miRNA; ns: not significant; *** p < 0.001; n = 5. (TIF) [file pone.0136285.s002.tif]

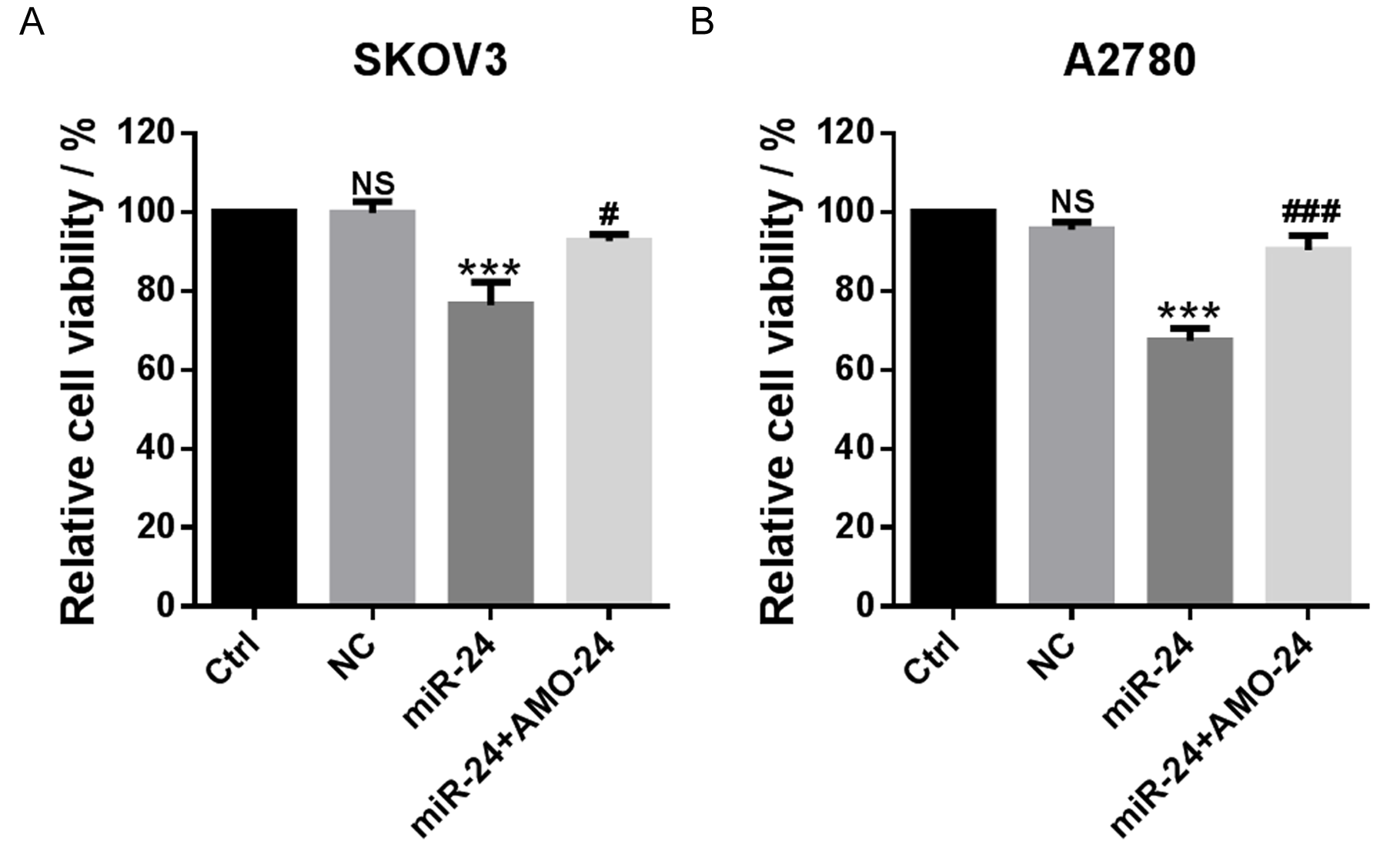

Supplement: S3 Fig — NC: negative control cells that were transfected with scramble miRNA; ns: not significant; *** p < 0.001 versus NC; # p < 0.05, ### p < 0.001 versus miR-24, n = 5. (TIF) [file pone.0136285.s003.tif]

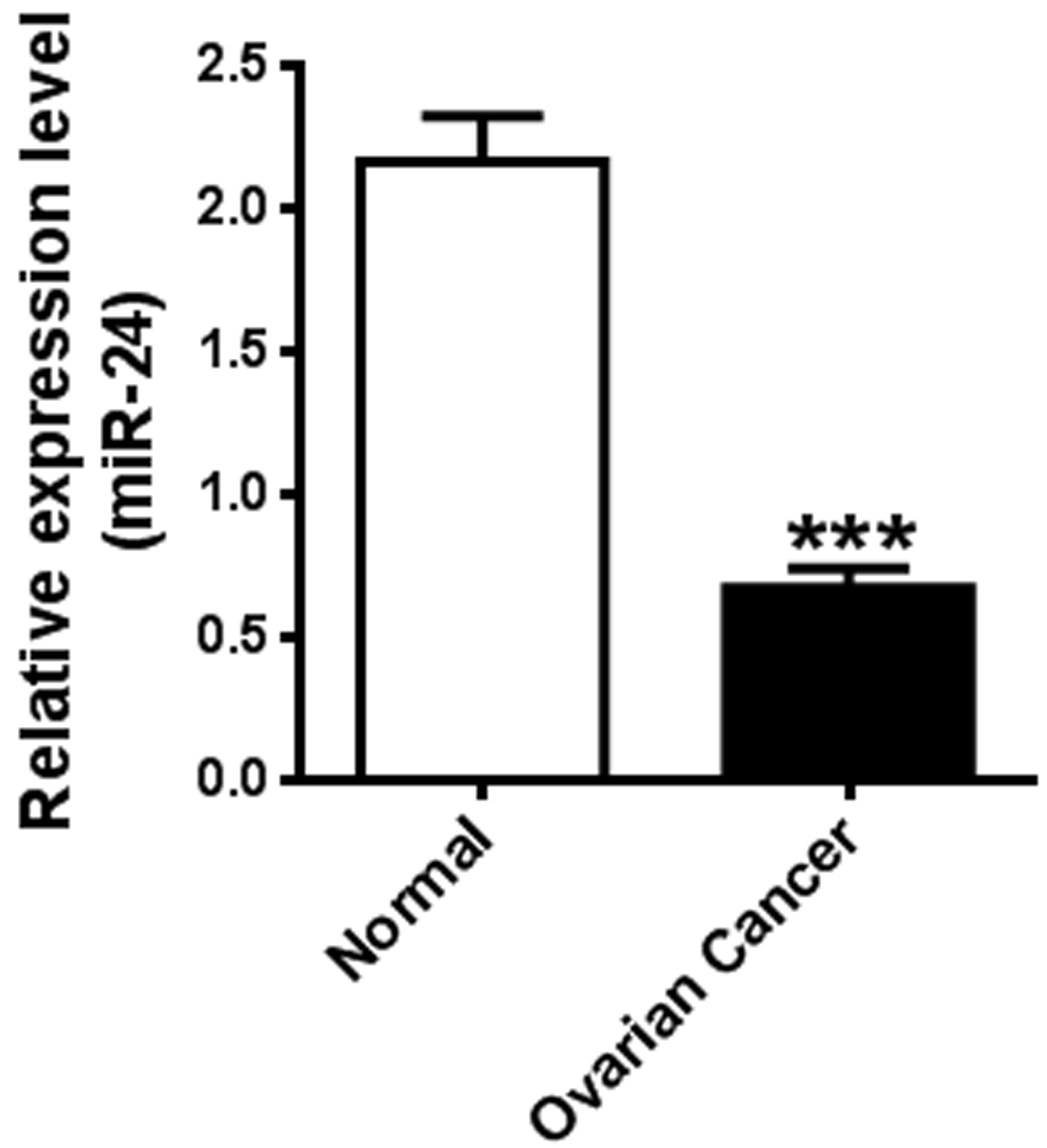

Supplement: S4 Fig — *** p < 0.001 versus normal. (TIF) [file pone.0136285.s004.tif]
